# Costing Methods Applied to Cancer

**DOI:** 10.1200/JGO.19.00222

**Published:** 2019-08-08

**Authors:** Susan Horton, Sumit Gupta

**Affiliations:** ^1^ University of Waterloo, Waterloo, Ontario, Canada; ^2^ The Hospital for Sick Children, Toronto, Ontario, Canada

Health care is vital in improving quality and length of life. However, because resources are scarce, there is considerable interest in costing of health care services, including studies of specific illnesses. These studies can be used for different purposes, including prioritization of health expenditures, deciding between different therapies for the same condition, or advocating for additional resources for conditions where the benefits of treatment (ie, reduction in future treatment costs, productivity benefits gained by prolonging survival of young people, or simply additional years of healthy life) outweigh the costs.

Herein, we discuss the methodological issues involved in costing interventions for individual diseases in low- and middle-income countries (LMICs), using a recently published article on treatment of a lymphoma in Malawi as an example.^1^ Our aim is to summarize best practices in costing to ensure that different studies produce comparable results. It is obviously important that authors of different costing studies (for different diseases in the same country, for the same disease in different countries, and so on) use similar methodologies to facilitate comparisons and avoid false conclusions. Similarly, health policymakers will be misled by costing studies that underestimate costs or omit key cost components.

Costing for different diseases can be used for different purposes. A recently developed reference case for costing for global health^2^ identifies four possible purposes for a costing study: economic evaluation, financial planning, budgeting, and efficiency analysis. It also identifies some key choices to make in structuring the analysis, including perspective (societal, payer, health system, and so on) and type of costs (incremental *v* full, economic *v* financial, and so on). It is frequently of interest to compare costs for different interventions, diseases, or countries. Thus it is especially important that the purpose of a particular costing study is well defined and costs are appropriately measured.

One important distinction is between top-down and bottom-up (ie, microcosting or ingredients-based) costing approaches. Top-down costing approaches may be used, for example, to allocate health spending by disease category, which was a focus of recent work by the Organisation for Economic Co-operation and Development.^3^ Top-down approaches aim to ensure that above-service and overhead costs of operating health facilities and health systems are not missed. Such costs include training, utilities, and administration, which may be of interest to health policymakers. In contrast, microcosting (ie, a bottom-up approach) is often used to examine efficiency (to compare different treatments for the same condition, treatment of the same condition in different countries or contexts, and so on). In microcosting, it is important not to miss key costs. It is frequently difficult to reconcile the sum of expenditures on all diseases obtained by bottom-up costing with total health spending. An Organisation for Economic Co-operation and Development study that aimed to allocate health spending by disease category (ie, International Classification of Diseases) across 15 countries found that bottom-up methods left significant amounts of unallocated expenditure compared with the national System of Health Accounts.^3^ The difference between top-down and bottom-up approaches is discussed further elsewhere.^4,5^

A recent systematic review that used mainly bottom-up methods of costing for pediatric cancer in LMICs identified 30 studies; leukemias and lymphomas were the most frequent subject (18 of the 30 studies), eight studies were for solid tumors, two of the studies compared two cancers, and two studies examined top-down costing of pediatric cancer centers/units (Fung et al, manuscript submitted for publication). The methodologies varied considerably, but many studies excluded key cost components. Only 11 of the studies included health care worker salaries, only 15 included patient accommodation, and only three considered administration costs (including both of the top-down studies). The omission of administration costs is a common pitfall. Hospitals are complex facilities with high overhead costs (utilities, space, human resource managers, legal and contract staff, personnel who record patient admissions or manage cancer registries, and so on). The costs of these shared services, which undoubtedly improve the quality of patient care, are hard to measure and allocate. Their omission, however, significantly underestimates cost.

Another methodological issue is whether cost should be calculated for a patient who has completed the regular treatment regimen or across all new diagnoses. This is particularly relevant in LMICs. For example, treatment abandonment rates in LMICs are significant and increase the cost per outcome (eg, survival or number of quality-adjusted life-years saved^6^). Reducing abandonment rates requires additional expenditures (including on social workers and the provision of hostels for caregivers) that are often partially covered by nongovernment organizations.^7^ Thus, costing out an idealized regimen without considering abandonment or expenditures to reduce abandonment is another way in which costs per patient are underestimated. Treatment-related mortality, which is also more common in LMICs, is another cause of early treatment failure that is ignored by costing idealized regimens.

The recently published article by Painschab et al^1^ extends the limited literature on costing of cancer treatment of adults in an LMIC. The authors adopt a microcosting methodology and state that they are using a health system perspective to answer the question, “How much does it cost to treat lymphoma in Malawi?” They made efforts to cover a variety of costs, including the time spent by medical personnel on procedures with the patient, as well as costs of diagnostic tests, treatment, and hospitalization. They also estimated some overhead costs, which were defined as the costs of setting up the pathology and laboratory infrastructure.

This is better than many other studies reviewed for pediatric cancer in LMICs (Fung et al, manuscript submitted for publication) but still far from the health system perspective desired by the authors. First, medical personnel do not spend every minute in contact with patients; they also spend time training others, being trained, doing necessary paperwork, attending meetings with colleagues, and so on. Arguably, this time is of value to patient care, even though it is not spent directly with patients. This so-called overhead time therefore needs to be allocated pro rata to each patient-contact minute. Second, it does not seem that the authors included the costs of essential nonmedical personnel involved in cancer care. Although the costs of some of these personnel (eg, janitors, cooks) may be subsumed in the bed-night costs, the costs of personnel who register patients, order supplies, and run a cancer registry, for example, are not. Moreover, central administration and utilities are not included. Third, costing an idealized average treatment pattern for a patient who has completed treatment and follow-up underestimates the cost per new diagnosis, because it omits the costs of those who abandon treatment or succumb to treatment complications.

Compared with many previously published studies, the Malawi study^1^ makes far greater efforts to include more comprehensive costs. Precisely which costs should be calculated depends on the purpose for which it is being used. If the aim is simply to compare costs of two different treatments in the same hospital, then it is acceptable to omit hospital overhead costs common to both treatments. However, the authors of the recent study^1^ are more ambitious but unfortunately do not answer their question (“How much does it cost to treat lymphoma in Malawi?”), and certainly not from a health system perspective. There are also too many omitted costs to use these estimates for cost-effectiveness calculations, which the authors correctly state are needed. We hope that the suggestions provided herein can help authors of similar studies (which are, indeed, badly needed) to continue to improve costing methodology.
